# Ginsenoside Re protects methamphetamine-induced dopaminergic neurotoxicity in mice via upregulation of dynorphin-mediated κ-opioid receptor and downregulation of substance P-mediated neurokinin 1 receptor

**DOI:** 10.1186/s12974-018-1087-7

**Published:** 2018-02-21

**Authors:** Duy-Khanh Dang, Eun-Joo Shin, Dae-Joong Kim, Hai-Quyen Tran, Ji Hoon Jeong, Choon-Gon Jang, Seung-Yeol Nah, Jung Hwan Jeong, Jae Kyung Byun, Sung Kwon Ko, Guoying Bing, Jau-Shyong Hong, Hyoung-Chun Kim

**Affiliations:** 1 Neuropsychopharmacology and Toxicology Program, College of Pharmacy, Kangwon National University, Chunchon, 24341 Republic of Korea; 2 Department of Anatomy and Cell Biology, Medical School, Kangwon National University, Chunchon, 24341 Republic of Korea; 3 Department of Pharmacology, College of Medicine, Chung-Ang University, Seoul, 06974 Republic of Korea; 4 Department of Pharmacology, School of Pharmacy, Sungkyunkwan University, Suwon, 16419 Republic of Korea; 5 Ginsentology Research Laboratory and Department of Physiology, College of Veterinary Medicine and Bio/Molecular Informatics Center, Konkuk University, Seoul, 05029 Republic of Korea; 6 Headquarters of Forestry Support, Korea Forestry Promotion Institute, Seoul, 07570 Republic of Korea; 7 Korean Society of Forest Environment Research, Namyangju, 12014 Republic of Korea; 8 Department of Oriental Medical Food and Nutrition, Semyung University, Jecheon, 27136 Republic of Korea; 9 Department of Anatomy and Neurobiology, University of Kentucky College of Medicine, 800 Rose Street, Lexington, KY 40536 USA; 10 Neuropharmacology Section, Laboratory of Toxicology and Pharmacology, National Institute of Environmental Health Sciences, Research Triangle Park, Durham, NC 27709 USA

**Keywords:** Methamphetamine, Dynorphin, κ-opioid receptor, Microglia, Neurokinin 1 receptor

## Abstract

**Background:**

We previously reported that ginsenoside Re (GRe) attenuated against methamphetamine (MA)-induced neurotoxicity via anti-inflammatory and antioxidant potentials. We also demonstrated that dynorphin possesses anti-inflammatory and antioxidant potentials against dopaminergic loss, and that balance between dynorphin and substance P is important for dopaminergic neuroprotection. Thus, we examined whether GRe positively affects interactive modulation between dynorphin and substance P against MA neurotoxicity in mice.

**Methods:**

We examined changes in dynorphin peptide level, prodynorphin mRNA, and substance P mRNA, substance P-immunoreactivity, homeostasis in enzymatic antioxidant system, oxidative parameter, microglial activation, and pro-apoptotic parameter after a neurotoxic dose of MA to clarify the effects of GRe, prodynorphin knockout, pharmacological inhibition of κ-opioid receptor (i.e., nor-binaltorphimine), or neurokinin 1 (NK1) receptor (i.e., L-733,060) against MA insult in mice.

**Results:**

GRe attenuated MA-induced decreases in dynorphin level, prodynorphin mRNA expression in the striatum of wild-type (WT) mice. Prodynorphin knockout potentiated MA-induced dopaminergic toxicity in mice. The imbalance of enzymatic antioxidant system, oxidative burdens, microgliosis, and pro-apoptotic changes led to the dopaminergic neurotoxicity. Neuroprotective effects of GRe were more pronounced in prodynorphin knockout than in WT mice. Nor-binaltorphimine, a κ-opioid receptor antagonist, counteracted against protective effects of GRe. In addition, we found that GRe significantly attenuated MA-induced increases in substance P-immunoreactivity and substance P mRNA expression in the substantia nigra. These increases were more evident in prodynorphin knockout than in WT mice. Although, we observed that substance P-immunoreactivity was co-localized in NeuN-immunreactive neurons, GFAP-immunoreactive astrocytes, and Iba-1-immunoreactive microglia. NK1 receptor antagonist L-733,060 or GRe selectively inhibited microgliosis induced by MA. Furthermore, L-733,060 did not show any additive effects against GRe-mediated protective activity (i.e., antioxidant, antimicroglial, and antiapoptotic effects), indicating that NK1 receptor is one of the molecular targets of GRe.

**Conclusions:**

Our results suggest that GRe protects MA-induced dopaminergic neurotoxicity via upregulatgion of dynorphin-mediated κ-opioid receptor and downregulation of substance P-mediated NK1 R.

**Electronic supplementary material:**

The online version of this article (10.1186/s12974-018-1087-7) contains supplementary material, which is available to authorized users.

## Background

Dynorphin is an endogenous opioid peptide that is widely distributed in various tissues. Numerous studies have suggested the involvement of dynorphin in the pathogenesis of Parkinson’s disease (PD). Earlier studies reported that reduced mRNA levels of prodynorphin were observed in the substantia nigra (SN) in postmortem brain specimens of Parkinsonian patients and animal models of PD [1, 2]. We reported that endogenous dynorphin attenuates dopaminergic neurotoxicity induced by 1-methyl-4-phenyl-1, 2, 3, 6-tetrahydropyridine (MPTP) or methamphetamine (MA) [3], and that genetic depletion of dynorphin potentiated induction of pro-inflammatory microglia of M1 phenotype (CD16, CD32, and CD86) [3].

Substance P is a potent pro-inflammatory neuropeptide with high concentrations in the SN [4, 5]. Previous reports have demonstrated that substance P receptor (neurokinin-1 receptor) antagonists prevented MA-induced loss of neurochemical markers of toxicity such as tyrosine hydroxylase, dopamine transporters, and tissue dopamine content in the striatum [6, 7]. Hong and colleagues [8–14] demonstrated the pathogenic role of microglia-induced neuroinflammation in multiple neurodegenerative disorders, including PD. Substance P is capable of stimulating microglial activation to produce superoxide and pro-inflammatory cytokines, thereby exacerbating dopaminergic neurodegeneration [13, 14]. We recently demonstrated that substance P might account for the increased density of microglia in the SN through chemotaxic recruitment via a novel NK1-NOX2 axis-mediated pathway [15].

Evidence has been reported which suggests the existence of a dopaminergic influence on the substance P striatonigral neurons [4, 16–19]. After chronic administration of the antipsychotic haloperidol, a potent dopamine receptor antagonist, nigral substance P-like immunoreactivity (SP-IR) was reduced substantially [20–22]. The enhancement of dopamine activity with repeated administrations of the indirect dopamine agonist methamphetamine (MA) causes elevations of SP-IR in several structures associated with the basal ganglia including SN [16]. This MA effect was blocked by concurrent administration of haloperidol, suggesting that the action of the drug on the striatonigral substance P neurons is mediated through the dopamine system [16]. Importantly, Block et al. [23] found that dynorphin inhibited substance P-induced dopaminergic neurotoxicity in vivo, suggesting the balance between dynorphin and substance P is essential for dopaminergic regulation.

Ginsenoside Re (GRe), a potential ginsenoside from Panax ginseng, exhibited different pharmacological activities via multiple mechanisms both in vivo and in vitro [24–29]. We reported that GRe rescues MA-induced dopaminergic degeneration and pro-inflammatory changes (i.e., activation of microglia) in vivo by inhibition of protein kinase Cδ [30]. Interestingly, our pilot study showed that GRe attenuated MA-induced decrease in prodynorphin mRNA expression in the striatum of wild-type mice [31].

To extend our knowledge, we investigated whether GRe requires dynorphin induction for protecting MA-induced dopaminergic toxicity. In addition, we also asked whether GRe modulates interaction between dynorphin/κ-opioid receptor and substance P/neurokinin 1 (NK1) receptor against MA toxicity. Here, we proposed that GRe-mediated neuroprotective effects in vivo against MA-induced dopaminergic toxicity require interactive modulation between dynorphin and substance P via upregulation of κ-opioid receptor and downregulation of NK1 receptor.

## Methods

### Animals

All animals were treated in accordance with the National Institutes of Health (NIH) Guide for the Humane Care and Use of Laboratory Animals (NIH Publication No. 85-23, 1985; grants.nih.gov/grants/olaw/references/PHSPolicyLabAnimals.pdf). The present study was performed in accordance with the Institute for Laboratory Research (ILAR) Guidelines for the Care and Use of Laboratory Animals. Mice were maintained under a 12-h light/12-h dark cycle and fed ad libitum. They were adapted to these conditions for 2 weeks prior to the experiment.

The prodynorphin knockout (DYN KO) mice were originally obtained by targeted deletion of the coding exons of the prodynorphin gene [32]. We used this animal model in our previous studies [33, 34]. The DYN KO strain used in the present study was backcrossed at least nine times to the C57BL/6 background. Prior to weaning, tail specimens were collected from each animal, and DNA was extracted to confirm the presence of the prodynorphin gene locus by polymerase chain reaction (PCR) using primer pairs specific for each genotype [32–34]. Primers to detect WT alleles at the prodynorphin gene locus were 5′-CAGGACCTGGTGCCGCCCTCAGAG-3′ and 5′-CGCTTCTGGTTGTCC CACTTCAGC-3′; primers specific for the deletion were 5′-ATCCAGGAAACCAGCAGCGGCTAT-3′ and 5′-ATTCAGACACATCCCACATAAGGACA-3′. The products were amplified in a GeneAmp PCR System 9700 (Applied Biosystems, Foster City, CA, USA) using the following PCR parameters: an initial denaturation at 94 °C for 5 min, and then 30 cycles of 94 °C for 30 s, 65 °C for 30 s, 72 °C for 30 s, and 72 °C for 5 min followed by electrophoresis on 1% agarose gels with ethidium bromide and photography under ultraviolet (UV) light.

### Drug treatment

Mice were treated with a single dose of MA (35 mg/kg, i.p.) or saline, and sacrificed 1 h, 3 h, 6 h, 12 h, 1 day, and 3 days after MA treatment to examine prodynorphin and substance P mRNA expressions, ROS formation, and HNE and protein carbonyl levels. High-performance liquid chromatography grade GRe with greater than 99% purity was provided by Dr. Sung Kwon Ko [35, 36]. GRe (20 mg/kg, i.p., twice a day) was given 5 days before and 1 day after MA injection. On the day of MA injection, GRe was administered at 2 h before and 10 h after MA injection. The dose of GRe was determined based on previous study [30]. Nor-binaltorphimine (Nor-B; κ-opioid receptor antagonist; Tocris Bioscience, Ellisville, MO, USA) was dissolved in sterile saline. Nor-B (3 or 6 mg/kg, i.p.) was given 3 h and 1.5 h before MA injection. The dose of Nor-B was determined based on previous study [34]. L-733,060 (NK1 receptor antagonist; Tocris Bioscience) was dissolved in sterile saline. L-733,060 (5 or 10 mg/kg, i.p.) was given 1 h before MA injection. The dose of L-733,060 was determined based on previous study [37].

### Dynorphin quantification

For sample preparation, brains were removed and placed into ice-cold oxygenated artificial cerebrospinal fluid (aCSF) for approximately 1 min before slicing. The dorsal striatum, NAc, and ventral midbrain were free-hand dissected from 400-μm-thick slices of brain tissue prepared using a vibrating tissue slicer. Tissue samples were placed in individual eppendorf tubes, flash-frozen in liquid nitrogen, and stored at − 80 °C until further use. All tissues were homogenized in RIPA buffer [150 mM NaCl, 1.0% Triton-X-100, 0.5% Sodium Deoxycholate, 0.1% Sodium Dodecyl Sulfate (SDS), 50 mM Trizma Base, pH 8.0] and centrifuged at 12,000×*g* for 30 min. The pellet was discarded and supernatant used in the enzyme-linked immunosorbant assay (ELISA). Protein was measured using the BCA protein assay reagent (Thermo Scientific, Rockford, IL, USA).

For enzyme-linked immunosorbant assay, a commercially available mouse-dynorphin ELISA kit (MBS727820; MyBioSource, Inc., San Diego, CA, USA) was utilized to determine the concentration of dynorphin in the tissue samples from WT mice. Briefly, standards and samples (5 μg protein/sample) were pipetted in duplicate into the 96-well plate provided. All reagents were added to wells, and the plate was washed and incubated according to kit instructions. The optical density of the samples was read within 4 min of the final incubation at 450 nm using a microplate reader (Molecular Devices Inc., Sunnyvale, CA, USA) [38].

### Tissue preparation for enzyme activity assays

Dorsal striatum, nucleus accumbens (NAc), and ventral midbrain were homogenized in 50 mM potassium phosphate buffer (pH 7.0) and centrifuged at 13,000 × *g* for 20 min. The resulting supernatant was used to measure the activities of glutathione peroxidase (GPx) and catalase. Additional dorsal striatum, NAc, and ventral midbrain were homogenized in 50 mM potassium phosphate buffer (pH 7.8) and were centrifuged at 13,000 × *g* for 20 min. The resulting supernatant was used to measure the activities of superoxide dismutase (SOD) [30].

### Determination of SOD activity

SOD activity was determined on the basis of inhibition of superoxide-dependent reactions as described previously [30] The reaction mixture contained 70 mM potassium phosphate buffer (pH 7.8), 30 μM cytochrome c, 150 μM xanthine, and tissue extract in phosphate buffer diluted tenfold with PBS in a final volume of 3 mL. The reaction was initiated by adding 10 μL of 50 units xanthine oxidase, and the change in absorbance at 550 nm was recorded. One unit of SOD was defined as the quantity required inhibiting the rate of cytochrome c reduction by 50%. For estimating total SOD, 10 μM potassium cyanide (KCN) was added to the medium to inhibit cytochrome oxidase activity.

### Determination of catalase activity

Catalase activity was determined by the rate of hydrogen peroxide absorbance decrease at 240 nm [30]. The reaction mixture contained 50 mM potassium phosphate buffer (pH 7.0) and an aliquot of the sample. The reaction started with adding hydrogen peroxide (final concentration of 10 mM), and absorbance was monitored at 25 °C for 5 min. Catalase from bovine liver (Sigma-Aldrich, St. Louis, MO, USA) was used as a standard.

### Determination of glutathione peroxidase (GPx) activity

GPx activity was analyzed by a spectrophotometric assay described by Shin et al. [30], using 2.0 mM reduced glutathione and 0.25 mM cumene hydroperoxide as substrates. The reaction rate at 340 nm was determined using the NADPH extinction coefficient (6.22 mM^− 1^ cm^− 1^). GPx activity was expressed as nanomole NADPH oxidized per minute per milligram protein at 25 °C. Protein was measured using the BCA protein assay reagent, and bovine serum albumin was used as a standard.

### Western blot analysis

Striatal tissues were lysed in buffer containing a 200 mM Tris–HCl (pH 6.8), 1% SDS, 5 mM ethylene glycol-bis (2-aminoethyl ether)-N, N, N′, N′-tetraacetic acid (EGTA), 5 mM EDTA, 10% glycerol, 1 × phosphatase inhibitor cocktail I (Sigma-Aldrich, St. Louis, MO, USA), and 1 × protease inhibitor cocktail (Sigma-Aldrich). Lysate was centrifuged at 12,000×*g* for 30 min, and the supernatant fraction was utilized for western blot analysis as described previously [30, 39]. Proteins (20 μg/lane) were separated by 8 or 10% SDS-PAGE, and transferred onto PVDF membranes. Following transfer, the membranes were preincubated with 5% non-fat milk for 30 min and were incubated overnight at 4 °C with primary antibody against NeuN (1:200; EMD Millipore, Temecular, MA, USA), GFAP (1:500; Santa Cruz Biotechnology, Santa Cruz, CA, USA), Iba-1 (1:500; Abcam, Cambridge, MA, USA), Bax (1:1000; Santa Cruz Biotechnology), cleaved caspase-3 (1:1000; Cell Signaling Technology, Inc., Danvers, MA, USA), caspase-3 (1:1000; Cell Signaling Technology, Inc.), Bcl-2 (1:1000; Santa Cruz Biotechnology), TH [1:5000; Chemicon (EMD Millipore)], or β-actin (1:50000; Sigma-Aldrich). Membranes were then incubated with HRP-conjugated secondary anti-rabbit IgG (1:1000, GE Healthcare, Piscataway, NJ, USA), anti-mouse IgG (1:1000, Sigma-Aldrich), or anti-goat IgG (1:1000, Sigma-Aldrich) for 2 h. Subsequent visualization was conducted using an enhanced chemiluminescence system (ECL plus®, GE Healthcare, Arlington Heights, IL, USA). Relative intensities of the bands were quantified by PhotoCapt MW (version 10.01 for Windows; Vilber Lourmat, Marne la Vallée, France) and then normalized to the intensity of β-actin [30].

### Immunocytochemistry

Immunocytochemistry was performed as described previously [30]. Mice were perfused transcardially with 50 mL of ice-cold PBS (10 mL/10 g body weight) followed by 4% paraformaldehyde (20 mL/10 g body weight). Brains were removed and stored in 4% paraformaldehyde overnight. A series of every sixth section (35 μm thickness, 210 μm apart) from striatum was selected and subjected to immunocytochemistry. Sections were blocked with PBS containing 0.3% hydrogen peroxide for 30 min and then incubated in PBS containing 0.4% Triton X-100 and 1% normal serum for 20 min. After a 48-h incubation with primary antibody against TH [1:500; Chemicon (EMD Millipore)], Iba-1 (1:500, Wako Pure Chemical Industries, Chuo-ku, Osaka, Japan), or substance P (1:500, a kind gift from Dr. Jau-Shyong Hong, National Institute of Environmental Health Sciences, USA; this antibody has been described elsewhere [15]). Sections were incubated with the biotinylated secondary antibody (1:1000; Vector Laboratories, Burlingame, CA, USA) for 1 h. The sections were then immersed in a solution containing avidin–biotin peroxidase complex (Vector Laboratories) for 1 h, and 3,3′-diaminobenzidine was utilized as the chromogen. Digital images were acquired under an upright microscope (BX51; Olympus, Tokyo, Japan) using an attached digital microscope camera (DP72; Olympus) and an IBM-compatible PC.

ImageJ software, version 1.47 (National Institutes of Health, Bethesda, MD, USA) was employed to measure the immunoreactivities of TH and Iba-1 in the striatum, or substance P in the SN as described previously [3, 15, 40, 41]. Briefly, images were subjected to background subtraction to correct for uneven background. The entire striatal region (for TH-immunoreactivity) or the rectangular region (350 μm × 260 μm, *w × h*, for Iba-1- or substance P-immunoreactivity) was drawn as the region of interest (ROI). Hue, saturation, and brightness threshold values were set in the “Adjust Color Threshold” dialog box to select the immunoreactive area, and then the mean density was measured.

### Double-labeled immunocytochemistry

Brain sections of 5 μm thickness obtained from wild-type (B6) mice were placed on the same slide and processed for immunostaining. Tissues were adhered on poly-L-lysine-precoated coverslips, were fixed in PBS-4% para-formaldehyde (PFA), and were permeabilized with 0.1% Triton X-100 in PBS for 15 min. After saturation with PBS-1% BSA, tissues were incubated for 40 min with the primary antibody and were incubated for 40 min with the secondary antibody as follows: primary antisera were as follows: rabbit anti-substance P ([15]; diluted 1:100), mouse anti-NeuN (1:100, Chemicon), goat anti-GFAP (1:100, Abcam), and mouse anti-Iba1 (1:100, Abcam). Secondary antibodies were goat anti-mouse IgG H&L (Alexa Fluor® 568; 1:100, Invitrogen, Carlsbad, CA, USA), goat anti-rabbit IgG H&L (FITC; 1:500, Abcam), and donkey anti-goat IgG H&L (Alexa Fluor® 546; 1:200, Invitrogen). Images of samples were recorded using an FV1000 confocal microscope (Olympus). To minimize bleed-through, each signal in double- or triple-stained samples was imaged sequentially. Images were processed in accordance with the Guidelines for Proper Digital Image Handling using ImageJ and/or Adobe Photoshop CS3 (Adobe, San Jose, CA, USA).

### Dissection of substantia nigra (SN)

Brains were rapidly removed and cut into 1 mm coronal sections on ice. The SN was punched using a fine Pasteur pipette. The SN was then easily identified and freely dissected. The total time for isolation of the SN was less than 3 min. The tissues were stored at − 70 °C until analysis [42–44].

### Reverse transcription and polymerase chain reaction (RT-PCR)

Expression of prodynorphin and substance P was assessed using quantitative RT–PCR to analyze mRNA levels, as described previously [45]. Total RNA was isolated from striatal or nigral tissues using an RNeasy Mini Kit (Qiagen, Valencia, CA, USA) according to the manufacturer’s instructions. Reverse transcription reactions were carried out using the RNA to cDNA EcoDry Premix (Clontech, Palo Alto, CA, USA) with a 1-h incubation at 42 °C. PCR amplification was performed using 25 cycles of denaturation at 94 °C for 1 min, annealing at 60 °C for 2 min, and extension at 72 °C for 1 min. The primer sequences and predicted product sizes for the amplified genes were as follows: prodynorphin (510 bp), 5′-CAG GAC CTG GTG CCG CCC TCA GAG-3′ (forward) and 5′-CGC TTC TGG TTG TCC CAC TTC AGC-3′ (reverse); substance P (194 bp), 5′- AAG CCT CAG CAG TTC TTT GGA T-3′ (forward) and 5′-GTT CTG CAT CGC GCT TCT TTC-3′ (reverse); and glyceraldehyde 3-phosphate dehydrogenase (GAPDH; 450 bp), 5′-ACC ACA GTC CAT GCC ATC-3′ (forward) and 5′-TCC ACC ACC CTG TTG CTG TA-3′ (reverse). PCR products were separated on 2% agarose gels containing ethidium bromide and visualized under ultraviolet light. Quantitative analysis of RNA was performed using PhotoCapt MW (version 10.01 for Windows; Vilber Lourmat).

### Synaptosome preparation

Striatal tissue was homogenized in 10 volume of ice-cold 0.32 mol/L sucrose and centrifuged at low speed (800×*g*, 12 min, 4 °C). This resulted in supernatant (S1) that was removed and centrifuged at high speed (22,000×*g*, 20 min, 4 °C) in order to yield pelleted synaptosomes. An aliquot of the re-suspended (P2) synaptosomal fraction was used for determination of ROS formation [39].

### Determination of ROS formation

ROS formation in the synaptosome was assessed by measuring the conversion from 2′,7′-dichlorofluorescin diacetate (DCFH-DA) to dichlorofluorescin (DCF) [46]. Synaptosomal fractions were added to a tube containing 2 mL of phosphate-buffered saline (PBS) with 10 nmol of DCFH-DA, dissolved in methanol. The mixture was incubated at 37 °C for 3 h, and then fluorescence was measured at 480 nm excitation and 525 nm emission. DCF was used as a standard.

### Determination of 4-hydroxynonenal (HNE)

The amount of lipid peroxidation was determined by measuring the level of 4-hydroxynonenal (HNE) using the OxiSelect™ HNE adduct ELISA kit (Cell Biolabs, Inc., San Diego, CA, USA) according to the manufacturer’s instructions. 100 μL of striatal homogenate at a protein concentration of 10 μg/mL was incubated in a 96-well protein binding plate at 4 °C overnight. After protein adsorption, HNE adducts in each well were labeled with HNE antibody followed by HRP-conjugated secondary antibody. Colorimetric development was then performed with substrate solution. Absorbance was recorded at 450 nm using a microplate reader (Molecular Devices Inc.), and an amount of HNE adduct in each sample was calculated from the standard curve of HNE-BSA [30].

### Determination of protein carbonyl

The extent of protein oxidation was assessed by measuring the content of protein carbonyl groups, which was determined spectrophotometrically with the 2,4-dinitrophenylhydrazine (DNPH)-labeling procedure [30] as described by Oliver [47]. The results are expressed as nmol of DNPH incorporated/mg protein based on the extinction coefficient for aliphatic hydrazones of 21 mM^− 1^ cm^− 1^. Protein was measured using the BCA protein assay reagent (Thermo Scientific, Rockford, IL, USA).

### Measurement of dopamine (DA) and its metabolites

Mice were sacrificed by cervical dislocation, and the brains were removed. The dorsal striatum, NAc, and ventral midbrain were dissected as above, were immediately frozen in liquid nitrogen, and were stored at − 70 °C before assays. Tissues were weighed, ultrasonicated in 10% perchloric acid, and centrifuged at 20,000×*g* for 10 min. The levels of DA and its metabolites DOPAC and HVA were determined by HPLC coupled with an electrochemical detector, as described previously [3, 30, 43]. Supernatant aliquots (20 μL) were injected into an HPLC equipped with a C18 column with 3 μm particle size (Waters). The mobile phase was comprised of 26 mL of acetonitrile, 21 mL of tetrahydrofuran, and 960 mL of 0.15 M monochloroacetic acid (pH 3.0) containing 50 mg/L of EDTA and 200 mg/mL of sodium octyl sulfate. The amount of DA was determined by comparison of peak areas of tissue samples with standard and was expressed in ng/g of wet tissue.

### Statistical analyses

Data were analyzed using IBM SPSS ver. 21.0 (IBM, Chicago, IL, USA). One-way analysis of variance (ANOVA) or two-way ANOVA was employed for statistical analyses. Post-hoc Fisher’s least significant difference (LSD) pairwise comparison tests were then conducted. *p* values < 0.05 were considered to be significant.

## Results

### MA-induced changes in dynorphin level, prodynorphin mRNA, and substance P mRNA expression in the dorsal striatum of wild-type (WT) mice

**Fig. 1:**
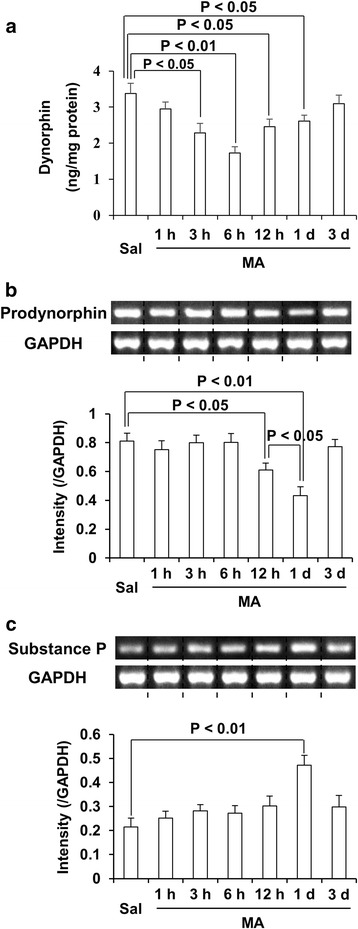
MA-induced changes in dynorphin level, prodynorphin mRNA, and substance P mRNA expression. Changes in dynorphin level (**a**), prodynorphin (**b**), and substance P (**c**) mRNA expression in the dorsal striatum of wild-type mice. Sal = saline, MA = methamphetamine 35 mg/kg, i.p. Each value is the mean ± SEM of six animals (one-way ANOVA followed by Fisher’s LSD pairwise comparisons)

We demonstrated a protective role of dynorphin in the modulation of dopaminergic system [3]. As shown in Fig. 1, we conducted a time-course study to elucidate changes in dynorphin level prodynorphin mRNA and substance P mRNA. Dynorphin level was significantly decreased 3 h (*p* < 0.05), 6 h (*p* < 0.01), 12 h (*p* < 0.05), and 1 day (*p* < 0.05) after a toxic dose of MA (Fig. 1a). The most pronounced decrease in dynorphin level was noted 6 h post-MA. Importantly, MA-induced decrease in dynorphin level in the dorsal striatum was most evident among nucleus accumbens, ventral midbrain, and dorsal striatum (please refer to Additional file 1: Figure S1). Interestingly, MA treatment did not change prodynorphin mRNA expression 3 and 6 h later, indicating that compensatory induction to significant decreases in dynorphin level. MA-induced significant decreases in prodynorphin mRNA expression was observed 12 h (*p* < 0.05) and 1 day (*p* < 0.01) later (Fig. 1b). This decrease was more pronounced in 1 day post-MA (*p* < 0.05) than in 12 h post-MA (Fig. 1b). As presented in Fig. 1c, a significant increase (*p* < 0.01) in substance P mRNA expression was noted 1 day after MA of WT mice. Therefore, we have focused on 1 day time-point for further study.

### Effects of genetic depletion of prodynorphin on MA-induced changes in superoxide dismutase (SOD), catalase, and glutathione peroxidase (GPx) activity in the dorsal striatum, nucleus accumbens (NAc), and ventral midbrain in mice

As shown in Table 1A–C, MA treatment initially increased (*p* < 0.05 vs. saline) SOD activity and decreased GPx activity 3 h later in the dorsal striatum (*p* < 0.05 vs. saline), NAc (*p* < 0.05 vs. saline) and ventral midbrain (*p* < 0.05 vs. saline) of WT mice. MA treatment initially increased (*p* < 0.05) catalase activity 6 h later in the ventral midbrain, however, initially increased catalase activity 3 h later in the dorsal striatum (*p* < 0.05 vs. saline) and NAc (*p* < 0.05 vs. saline) of WT mice. All of these changes returned near saline level 3 days later. These changes appeared to be more pronounced in dorsal striatum than in NAc or ventral midbrain.

**Table 1:** Effect of genetic depletion of prodynorphin on MA-induced changes in activity in enzymatic antioxidants

A								
SOD (units/mg protein)		Saline	MA					
			1 h	3 h	6 h	12 h	1 day	3 days
Dorsal striatum	WT	6.1 ± 0.6	7.5 ± 0.5^†^	8.4 ± 0.7^*’‡^	12 ± 0.4^**‡^	11.1 *±* 0.8^**‡^	10.0 ± 0.3^**‡^	6.5 ± 0.4
	DYN KO	6.0 ± 0.3	–	–	15.5 ± 0.7^**,#^	–	12.9 ± 0.3^**,#^	–
NAc	WT	5.0 ± 0.3	5.6 ± 0.5	6.7 ± 0.3^*^	6.3 ± 0.3^**^	7.1 ± 0.6^*^	6.8 ± 0.4^*^	5.7 ± 0.4
	DYN KO	4.8 ± 0.4	–	–	11.1 ± 0.5^**,#^	–	9.1 ± 0.5^**,#^	–
Ventral midbrain	WT	5.8 ± 0.4	6.5 *±* 0.4	7.4 *±* 0.3^*,&^	9.7 *±* 0.6^**,&^	8.4 ± 0.5^**,&^	7.9 *±* 0.4^**,&^	6.3 *±* 0.5
	DYN KO	5.9 *±* 0.6	*–*	*–*	12 *±* 0.6^**,#^	*–*	10.2 *+* 0.4	*–*
B								
Catalase (units/mg protein)		Saline	MA					
			1 h	3 h	6 h	12 h	1 day	3 days
Dorsal striatum	WT	1.7 ± 0.09	2.0 ± 0.16^†^	2.1 ± 0.07^*,†^	2.4 ± 0.14^**,†^	2.6 ± 0.19^**,‡^	2.5 ± 0.13^**,‡^	1.9 ± 0.09
	DYN KO	1.6 ± 0.08	–	–	2.4 ± 0.06^**^	–	2.2 ± 0.08^*^	–
NAc	WT	1.4 ± 0.06^&^	1.5 ± 0.1	1.6 ± 0.08^*^	1.7 ± 0.08	1.9 *±* 0.06^**^	1.8 ± 0.11^*^	1.6 ± 0.07^*^
	DYN KO	1.2 ± 0.05	–	–	1.8 ± 0.08^*^	–	1.6 ± 0.09^*^	–
Ventral midbrain	WT	1.6 ± 0.09	1.7 ± 0.32	1.8 ± 0.12	1.9 ± 0.09^*^	2.2 ± 0.13^**,&^	2.1 ± 0.11^**,&^	1.8 ± 0.09
	DYN KO	1.3 ± 0.07	–	–	2 ± 0.09^*^	–	1.8 ± 0.09^*^	–
C								
GPx (nmole NADPH/min/mg protein)		Saline	MA					
			1 h	3 h	6 h	12 h	1 day	3 days
Dorsal striatum	WT	9.9 + 0.3	8.5 ± 0.8	7.4 ± 0.4^*^	4.9 ± 0.5^**,†^	5.4 ± 0.3^**,†^	5.6 ± 0.4^**,†^	8.4 ± 0.6
	DYN KO	9.3 ± 0.5	–	–	3.9 ± 0.5^**,#^	–	5.3 ± 0.4^**,#^	–
NAc	WT	8.7 ± 0.4	8.3 ± 0.3	7.3 ± 0.3^*^	6.3 ± 0.3^**^	6.7 ± 0.2^**^	7.1 ± 0.5^**^	8.3 ± 0.2
	DYN KO	8.2 ± 0.4	–	–	4.9 ± 0.3^**,#^	–	6.1 ± 0.4^**,#^	–
Ventral midbrain	WT	9.2 ± 0.6	8.7 ± 0.3	7.8 ± 0.5^*^	6.7 ± 0.3^**^	6.7 ± 0.5^**^	7.0 ± 0.4^**^	8.6 ± 0.3
	DYN KO	8.9 ± 0.4	–	–	5.1 ± 0.5^**,#^	–	6.3 ± 0.5^**,#^	–

*SOD* superoxide dismutase, *GPx* glutathione peroxidase, *Sal* saline, *MA* methamphetamine 35 mg/kg, i.p, *WT* wild-type mice, *DYN KO* prodynorphin knockout mice. Each value is the mean ± SEM of six animals [two-way ANOVA (time points × brain regions) followed by Fisher’s LSD pairwise comparisons. Additional two-way ANOVA (time points × gene) with Fisher’s LSD pairwise comparisons was done to examine the effect of DYN KO 6 h and 1 day after MA]. ^*^p < 0.05,^**^p < 0.01 vs. corresponding Saline.^#^*p* < 0.05,^##^*p* < 0.01 vs. corresponding MA/WT. ^†^*p* < 0.05, ^‡^*p* < 0.01 vs. corresponding NAc or ventral midbrain. ^&^*p* < 0.05 vs. corresponding NAc

In addition, MA treatment exhibited in a sustained increases in SOD activity 6 h (dorsal striatum, NAc, or ventral midbrain; *p* < 0.01 vs. corresponding saline), 12 h (dorsal striatum or ventral midbrain; *p* < 0.01 vs. corresponding saline. NAc; *p* < 0.05 vs. corresponding saline), and 1 day (dorsal striatum or ventral midbrain; *p* < 0.01 vs. corresponding saline. NAc; *p* < 0.05 vs. corresponding saline) later in WT mice. SOD activity of dorsal striatum (SOD activity 1 h, 3 h, 12 h, or 1 day post-MA; *p* < 0.05 vs. corresponding SOD activity of NAc or ventral midbrain) was significantly higher than that of NAc or ventral midbrain. SOD activity of ventral midbrain (3 h, 6 h, 12 h, or 1 day post-MA; *p* < 0.05 vs. corresponding SOD activity of NAc) was higher than that of NAc (Table 1A). We examined whether genetic depletion of prodynorphin affects enzymatic antioxidants activity induced by MA. Because SOD and GPx activities were significantly changed 6 h post-MA and prodynorphin and substance P mRNA expressions were significantly changed 1 day post-MA, we focused on 6 h and 1 day post-MA.

Prodynorphin knockout significantly potentiated increases in SOD activity 6 h (dorsal striatum, NAc, or ventral midbrain; *p* < 0.05 vs. corresponding brain region of WT mice) and 1 day (dorsal striatum, NAc, or ventral midbrain; *p* < 0.01 vs. corresponding brain region of WT mice. Dorsal striatum; *p* < 0.05 vs. corresponding brain region of prodynorphin knockout mice) after MA treatment in WT mice (Table 1A). Similar to SOD, MA treatment significantly increased catalase activity in WT mice (Table 1B). However, genetic inhibition of prodynorphin did not significantly affect catalase activity of dorsal striatum, NAc, and ventral midbrain of WT mice (Table 1B).

In contrast, treatment with MA resulted in significant decreases in GPx activity 3 h (dorsal striatum, NAc, or ventral midbrain; *p* < 0.05 vs. corresponding saline), 6 h (dorsal striatum, NAc, or ventral midbrain; *p* < 0.01 vs. corresponding saline), 12 h (dorsal striatum, NAc, or ventral midbrain; *p* < 0.01 vs. corresponding saline), and 1 day (dorsal striatum, NAc, or ventral midbrain; *p* < 0.01 vs. corresponding saline) later in WT mice. GPx activity of dorsal striatum (6 h, 12 h, or 1 day post-MA; *p* < 0.05 vs. corresponding GPx activity of NAc or ventral midbrain) was significantly lower than that of NAc and ventral midbrain of MA-treated WT mice (Table 1C). Prodynorphin knockout significantly decreased (dorsal striatum, NAc, or ventral midbrain; *p* < 0.05 vs. corresponding brain region of WT mice) GPx activity 6 h and 1 day post-MA treatment in WT mice (Table 1C).

### MA-induced changes oxidative burdens [i.e., reactive oxidative stress (ROS), 4-hydroxynonenal (HNE), and protein carbonyl level] in the dorsal striatum of wild-type (WT) mice

**Fig. 2:**
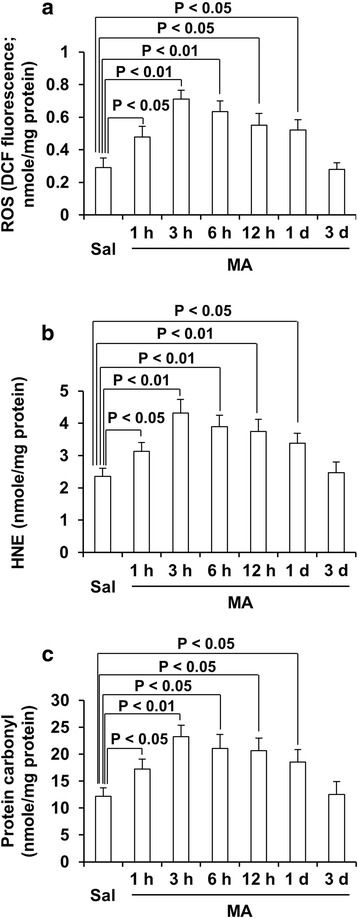
MA-induced changes oxidative burdens in the dorsal striatum of wild-type (WT) mice. Sal = saline, MA = methamphetamine 35 mg/kg, i.p., ROS = reactive oxygen species, HNE = 4-hydroxynonenal. Each value is the mean ± SEM of six animals (one-way ANOVA followed by Fisher’s LSD pairwise comparisons)

**Fig. 3:**
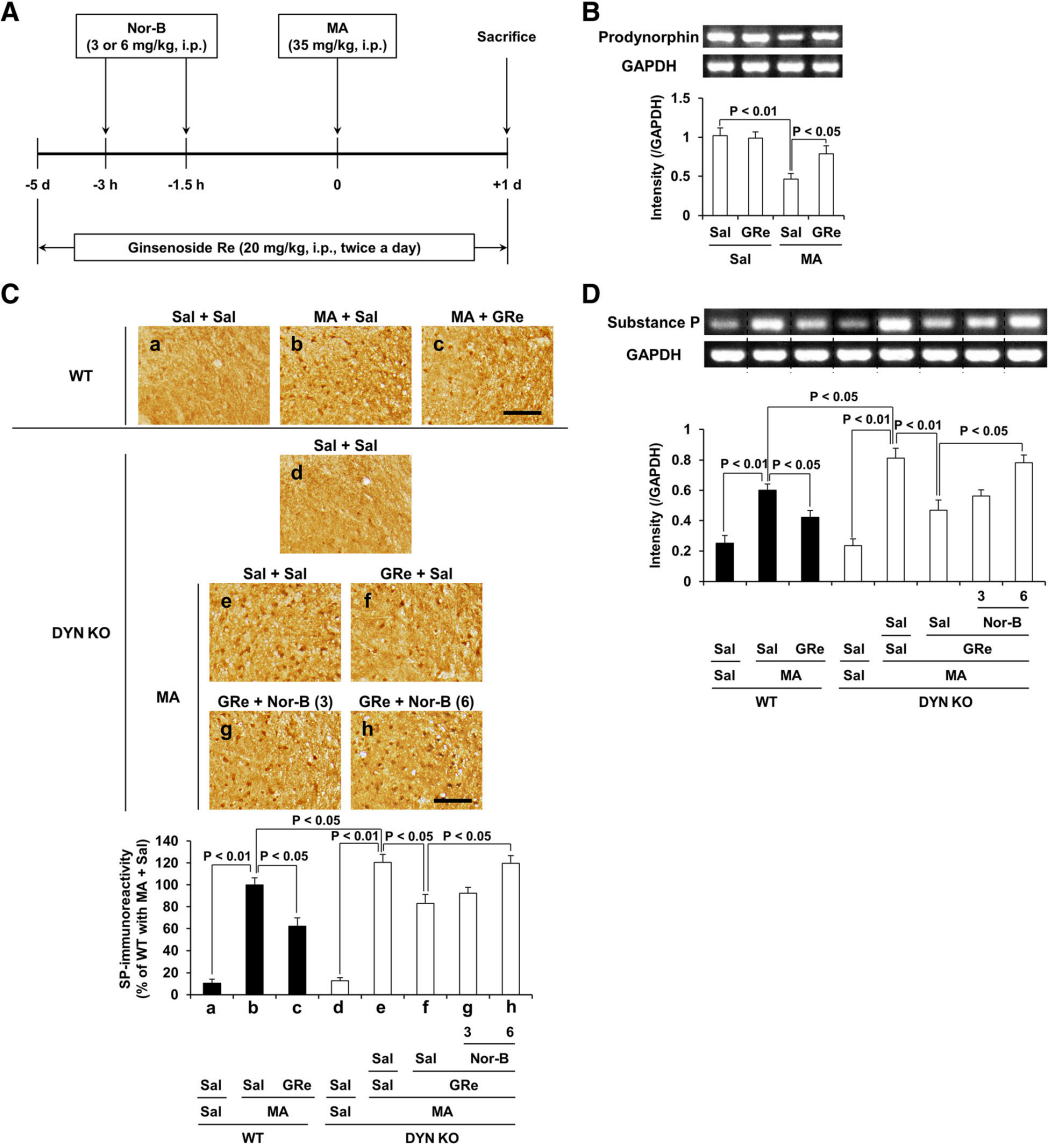
Role of κ-opioid receptor in GRe-mediated modulation of prodynorphin and substance P. Experimental design for determining whether κ-opioid receptor is involved in GRe-mediated effects against dopaminergic toxicity induced by MA; mice received Nor-B by two times (3 and 1.5 h) before MA treatment. Mice received ginsenoside Re twice a day for seven consecutive days (i.e., 5 days before and 1 day after MA) (**a**). Effect of GRe on MA-induced decrease in prodynorphin mRNA expression in the striatum of mice (**b**). Effects of κ-opioid receptor antagonist Nor-B on GRe-mediated pharmacological activity against MA-induced increases in substance P (SP)-immunoreactivity (**c**) and substance P mRNA expression (**d**) in the substantia nigra (SN) of DYN KO mice. Sal = saline, MA = methamphetamine 35 mg/kg, i.p., GRe = ginsenoside Re 20 mg/kg, i.p., Nor-B = nor-binaltorphimine 3 or 6 mg/kg, i.p., WT = wild-type mice, DYN KO = prodynorphin knockout. Each value is the mean ± SEM of six animals [two-way ANOVA (B) or one-way ANOVA (C and D) followed by Fisher’s LSD pairwise comparisons]. Scale bar = 100 μm

It is well-known that oxidative stress is a key element in MA neurotoxicity [48]. Because dorsal striatum was the most sensitive to MA-induced changes in enzymatic antioxidant as shown in Fig. 2, we focused on dorsal striatum. In our study, parameters of oxidative stress (i.e., ROS, HNE, and protein carbonyl level) were significantly elevated after MA (ROS: 1 h, *p* < 0.05; 3 h, *p* < 0.01; 6 h, *p* < 0.01; 12 h, *p* < 0.05; 1 day, *p* < 0.05. HNE: 1 h, *p* < 0.05; 3 h, *p* < 0.01; 6 h, *p* < 0.01; 12 h, *p* < 0.01; 1 day, *p* < 0.05. Protein carbonyl: 1 h, *p* < 0.05; 3 h, *p* < 0.01; 6 h, *p* < 0.05; 12 h, *p* < 0.05; 1 day, *p* < 0.05), which consistently reached the highest level 3 h post-MA (Fig. 3a–c).

### Effects of κ-opioid receptor antagonist nor-binaltorphimine (Nor-B) on the pharmacological activity of ginsenoside Re (GRe) against MA-induced increase in substance P level in WT and prodynorphin KO mice

As shown in Fig. 3a, Nor-B was treated two times before MA administration, and mice received GRe (20 mg/kg, i.p.) for 6 days (5 days before and 1 day after MA). As presented in Fig. 3b, MA-induced decrease (*p* < 0.01 vs. Sal/Sal) in prodynorphin mRNA expression was significantly attenuated (*p* < 0.05) by treatment with GRe. A little substance P-immunoreactivity was observed in the striatum of mice with or without MA (Additional file 1: Figure S2). However, there was a significant induction of substance P-immunoreactivity in the SN of WT mice 1 d post-MA (*p* < 0.01) (Additional file 1: Figure S2). GRe treatment attenuated MA-induced increases in the substance P-immunoreactivity (*p* < 0.05) and substance P mRNA expression (*p* < 0.05) in the SN of mice (Fig. 3c, d). MA-induced increases in substance P levels were more prominent in prodynorphin KO than WT mice (*p* < 0.05). Nor-B (6 mg/kg, i.p.) significantly counteracted GRe-mediated attenuation against substance P-immunoreactivity (*p* < 0.05) or substance P mRNA expression (*p* < 0.05) induced by MA in prodynorphin KO mice (Fig. 3c, d), indicating that GRe requires upregulation of κ-opioid receptor modulation for exerting antioxidant potential.

For more details on Nor-B activity against GRe-mediated effects in WT mice, please refer to Additional file 1: Figure S3.

### Effects of neurokinin 1 (NK1) receptor antagonist L-733,060 on κ-opioid receptor antagonist, nor-binaltorphimine (Nor-B)-mediated pharmacological activity in response to antioxidant effects of ginsenoside Re (GRe) against MA insult in the striatum of prodynorphin KO mice

**Fig. 4:**
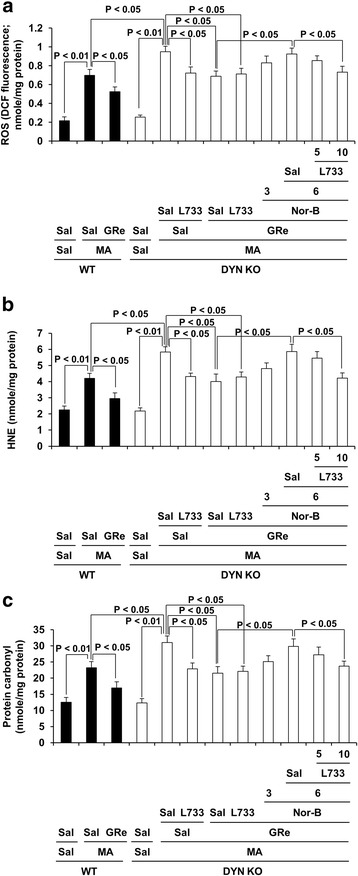
Role of κ-opioid receptor and neurokinin 1 receptor in GRe-mediated antioxidant potentials. Effects of neurokinin 1 receptor antagonist L-733,060 on Nor-B-mediated pharmacological activity in response to antioxidant effect of GRe against ROS formation (**a**), HNE (**b**), and protein carbonyl (**c**) levels induced by MA. Sal = saline, MA = methamphetamine 35 mg/kg, i.p., GRe = ginsenoside Re 20 mg/kg, i.p., Nor-B = Nor-binaltorphimine 3 or 6 mg/kg, i.p., L733 = L-733,060 5 or 10 mg/kg, i.p., ROS = reactive oxygen species, HNE = 4-hydroxynonenal, WT = wild type, DYN KO = prodynorphin knockout. Each value is the mean ± SEM of six animals (one-way ANOVA followed by Fisher’s LSD pairwise comparisons)

As shown in Fig. 4, oxidative stress (i.e., ROS, HNE, and protein carbonyl level) 3 h post-MA was more evident in prodynorphin KO mice (ROS, HNE, or protein carbonyl level: *p* < 0.05 vs. corresponding WT) than that in WT mice. Nor-B counteracted (ROS, HNE, or protein carbonyl level: *p* < 0.05, respectively) antioxidant effects of GRe in a dose-related manner. L-733,060 inhibited Nor-B-mediated counteraction against the antioxidant effect of GRe in prodynorphin KO mice (*p* < 0.05). In addition, L-733,060 itself attenuated against oxidative stress (ROS, HNE, or protein carbonyl level: *p* < 0.05, respectively) induced by MA. L-733,060 did not exhibit any additive effects in response to GRe-mediated antioxidant effect, suggesting that GRe possesses an interactive pharmacological activity between κ-opioid receptor and NK1 receptor.

The effects of Nor-B on GRe-mediated antioxidant potential in WT mice were shown in Additional file 1: Figure S4.

### Substance P-immunoreactivity is expressed in NeuN-immunoreactive neurons, GFAP-immunoreactive astrocytes, or Iba-1-immunoreactive microglial cells in the substantia nigra of WT mice

**Fig. 5:**
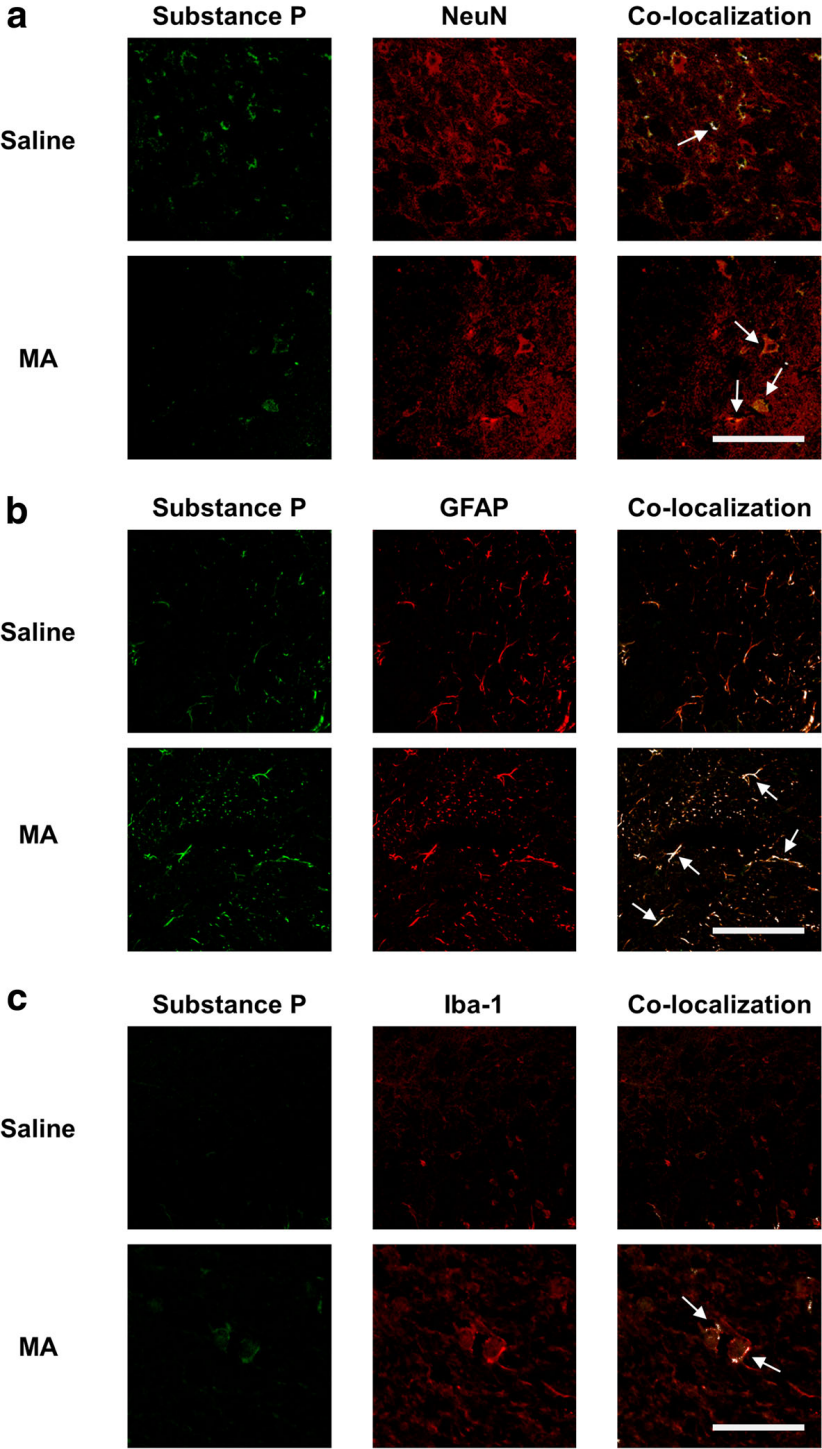
MA-induced changes in substance P-immunodistribution. Substance P-immunoreactivity was localized in the NeuN-labeled neurons (**a**), GFAP-labeled astrocytes (**b**), and Iba-1-labeled microglial cells (**c**). Arrow indicates co-localization. Scale bar = 100 μm

It was also reported that microglia expresses the substance P gene and NK1 receptor [49]. In order to determine if substance P modulates neuron, astrocyte, and microglia in our experimental system, we conducted double-labeling immunocytochemistry. Substance P-immunoreactivity was co-localized in NeuN-, GFAP-, or Iba-1 immunoreactive cells, suggesting that substance P-immunoreactivity is expressed in neurons, reactive astrocytes, or reactive microglia after MA treatment (Fig. 5).

### Effects of neurokinin 1 (NK1) receptor antagonist, L-733,060 on κ-opioid receptor antagonist, nor-binaltorphimine (Nor-B)-mediated pharmacological activity in response to effects of ginsenoside Re (GRe) against changes in NeuN, GFAP, and Iba-1 expression induced by MA in the striatum of prodynorphin KO mice

**Fig. 6:**
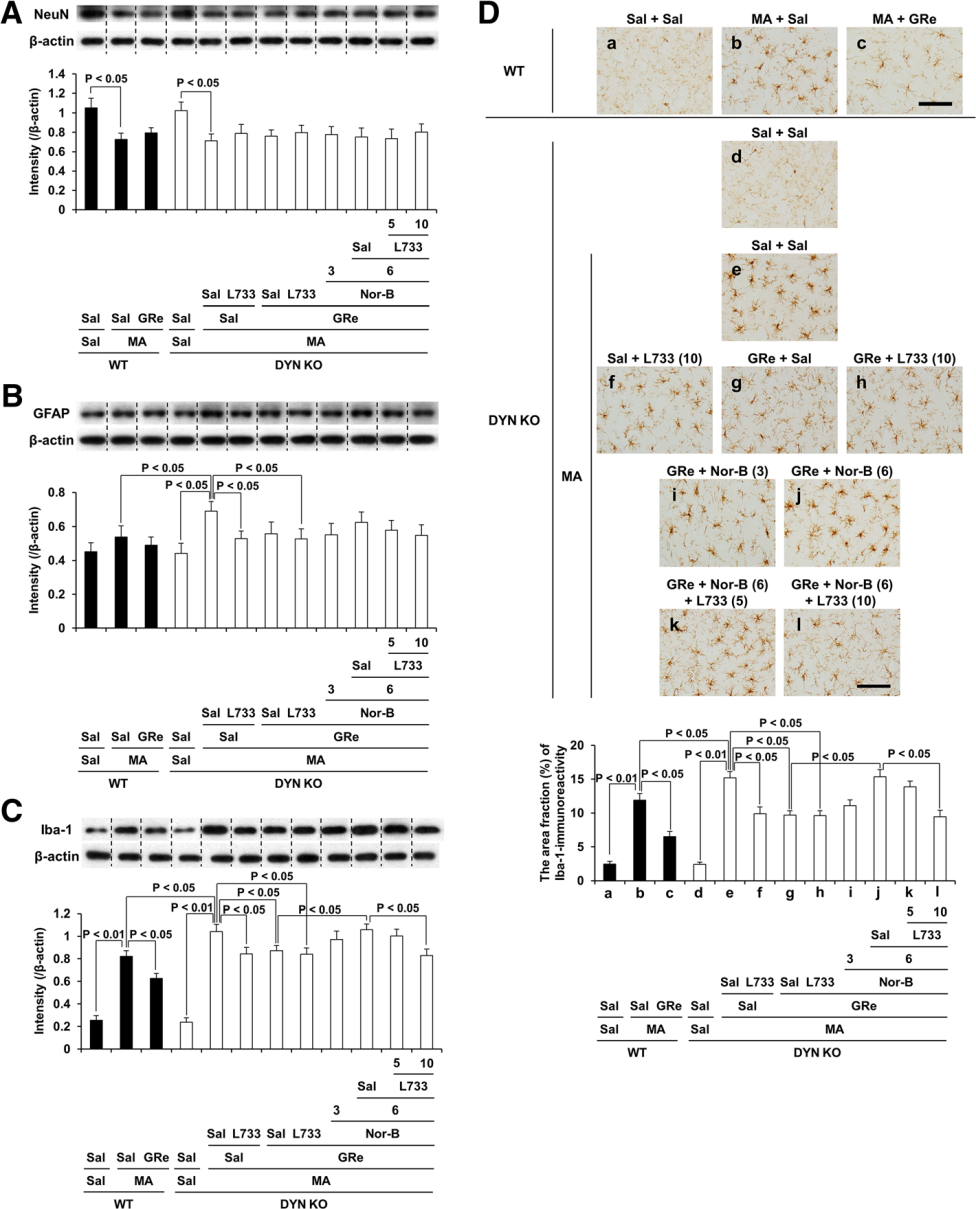
Role of κ-opioid and neurokinin 1 receptors in GRe-mediated neuronal, astrocytic, and microglial modulation. Effects of neurokinin 1 receptor antagonist L-733,060 on κ-opioid receptor antagonist Nor-B-mediated pharmacological activity in response to effects of GRe against the changes in NeuN (**a**), GFAP (**b**), Iba-1 expression (**c**), and Iba-1-immunoreactivity (**d**) induced by MA in the striatum of DYN KO mice. Sal = saline, MA = methamphetamine 35 mg/kg, i.p., GRe = ginsenoside Re 20 mg/kg, i.p., Nor-B = Nor-binaltorphimine 3 or 6 mg/kg, i.p., L733 = L-733,060 5 or 10 mg/kg, i.p., GFAP = glial fibrillary acidic protein, Iba-1 = ionized calcium-binding adapter molecule 1, WT = wild type, DYN KO = prodynorphin knockout. Each value is the mean ± SEM of six animals (one-way ANOVA followed by Fisher’s LSD pairwise comparisons). Scale bar = 100 μm

As presented in Fig. 6a, MA treatment significantly decreased (*p* < 0.05) NeuN expression in both WT and prodynorphin KO mice. GRe, L-733,060, or Nor-B did not significantly alter NeuN expression induced by MA. In contrast, MA tended to increase GFAP expression without reaching statistical analysis in WT mice. However, MA significantly increased (*p* < 0.05) GFAP expression in DYN KO mice. MA-induced increase in GFAP expression was prevented (*p* < 0.05) by L-733,060, but not by GRe (Fig. 6b). Importantly, Iba-1 expression was significantly increased in the striatum 1 day after MA (*p* < 0.05). MA-induced increase in Iba-1 expression was more evident (*p* < 0.05) in prodynorphin KO than in WT mice. Either GRe (*p* < 0.05) or L-733,060 (*p* < 0.05) significantly attenuated MA-induced increase in Iba-1 expression. Treatment of L-733,060 did not exhibit any additional effects in response to the pharmacological activity of GRe. Nor-B (6 mg/kg, i.p.) counteracted (*p* < 0.05) against GRe-mediated attenuation in Iba-1 expression. L-733,060 treatment inhibited (*p* < 0.05) Nor-B-induced counteraction (Fig. 6a). This phenomenon consistently paralleled Iba-1-immunoreactivity (Iba-1-IR), indicating that western blotting analysis (Fig. 6c) supports immunostaining (Fig. 6d). As shown in Additional file 1: Figure S5, MA-induced Iba-1-immunoreactive microglial cells were more pronounced in the striatum than in the SN. For more details on the effects of Nor-B on the protective activity of GRe against microglial activation induced by MA in WT mice, please refer to Additional file 1: Figure S6.

### Effects of neurokinin 1 (NK1) receptor antagonist L-733,060 on κ-opioid receptor antagonist nor-binaltorphimine (Nor-B)-mediated pharmacological activity in response to effects of ginsenoside Re (GRe) against pro-apoptotic changes induced by MA in the striatum of prodynorphin KO mice

Cadet and colleagues [50] made the first in vivo demonstration that a neurotoxic dose of MA causes differential regulation of several Bcl-2 family genes with two distinct clustering consisting of upregulation of pro-death and downregulation of anti-death gene expression. Their results brought further highlight the role that pro-apoptotic cell death plays an important role in MA neurotoxicity [51–57].

**Fig. 7:**
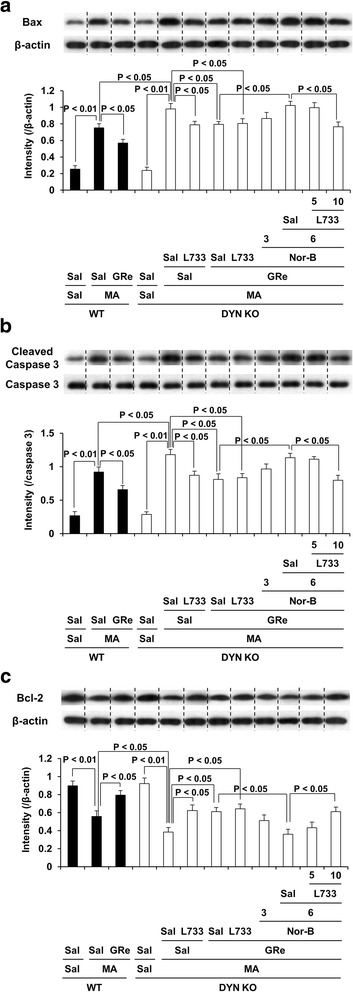
Role of κ-opioid and neurokinin 1 receptors in GRe-mediated anti-apoptotic potentials. Effects of neurokinin 1 receptor antagonist L-733,060 on κ-opioid receptor antagonist Nor-B-mediated pharmacological activity in response to effects of GRe against the changes in Bax (**a**), cleaved caspase-3 (**b**), and Bcl-2 expression (**c**) induced by MA in the striatum of DYN KO mice. Sal = saline, MA = methamphetamine 35 mg/kg, i.p., GRe = ginsenoside Re 20 mg/kg, i.p., Nor-B = Nor-binaltorphimine 3 or 6 mg/kg, i.p., L733 = L-733,060 5 or 10 mg/kg, i.p., WT = wild type, DYN KO = prodynorphin knockout. Each value is the mean ± SEM of six animals (one-way ANOVA followed by Fisher’s LSD pairwise comparisons)

As presented in Fig. 7a, b, MA treatment significantly increased Bax (*p* < 0.01) and cleaved caspase-3 (*p* < 0.01) expression in WT mice. MA-induced Bax and cleaved caspase-3 expression was more evident (*p* < 0.05, respectively) in prodynorphin KO mice than in WT. GRe or L-733,060 attenuated MA-induced increase in Bax (*p* < 0.05) and cleaved caspase-3 (*p* < 0.05) expression in prodynorphin KO mice. Consistently, MA significantly decreased (*p* < 0.01) Bcl-2 expression (Fig. 7c). This decrease was more pronounced in prodynorphin KO (*p* < 0.05) than in WT mice, which was significantly attenuated by treatment with GRe (*p* < 0.05) or L-733,060 (*p* < 0.05). L-733,060 did not produce any additional effect against the anti-apoptotic potential mediated by GRe. The attenuation by GRe was significantly counteracted (*p* < 0.05) by Nor-B (6 mg/kg, i.p.), which was subsequently inhibited (*p* < 0.05) by L-733,060 (10 mg/kg, i.p.).

The effects of Nor-B on the protective activity of GRe against MA-induced apoptotic changes in WT were presented in Additional file 1: Figure S7.

### Effects of genetic depletion of prodynorphin on MA-induced changes in dopamine and dopamine turnover rate in the dorsal striatum, nucleus accumbens (NAc), and ventral midbrain of mice

**Fig. 8:**
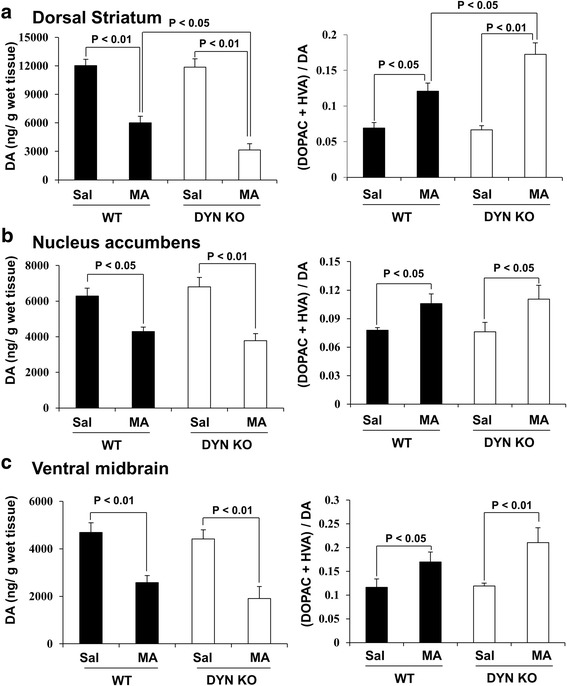
Effects of genetic depletion of DYN on MA-induced dopaminergic change. **a** Effects of dorsal striatum. **b** Effects of nucleus accumbens. **c** Effects of ventral midbrain. Sal = saline, MA = methamphetamine 35 mg/kg, i.p., GRe = ginsenoside Re 20 mg/kg, i.p., Nor-B = Nor-binaltorphimine 3 or 6 mg/kg, i.p., L733 = L-733,060 5 or 10 mg/kg, i.p., WT = wild type, DYN KO = prodynorphin knockout. Each value is the mean ± SEM of six animals (two-way ANOVA followed by Fisher’s LSD pairwise comparisons)

As shown in Fig. 8a–c, we investigated whether prodynorphin knockout affects MA-induced dopaminergic impairments in mice. MA-induced significant decreases in dopamine level was observed in dorsal striatum (*p* < 0.01 vs. saline), NAc (*p* < 0.05 vs. saline), and ventral midbrain (*p* < 0.01 vs. saline) in WT mice. MA-induced dopamine loss was more pronounced (*p* < 0.05 vs. WT) than in the dorsal striatum of prodynorphin knockout mice. However, prodynorphin knockout did not significantly affect MA-induced dopaminergic loss in NAc and ventral midbrain. Consistently, MA-induced dopamine turnover rate was significantly increased in dorsal striatum, NAc, and ventral midbrain in WT and prodynorphin knockout mice. Dopamine turnover rate was higher in the dorsal striatum of MA-treated prodynorphin knockout (*p* < 0.05) than WT mice. However, prodynorphin knockout did not significantly change dopamine turnover rate of NAc and ventral midbrain in mice. Thus, we focused on dorsal striatum for the mechanistic study on dopaminergic system.

### Effects of neurokinin 1 (NK1) receptor antagonist L-733,060 on κ-opioid receptor antagonist nor-binaltorphimine (Nor-B)-mediated pharmacological activity in response to effects of ginsenoside Re (GRe) against dopaminergic impairments induced by MA in the dorsal striatum of prodynorphin KO mice

**Fig. 9:**
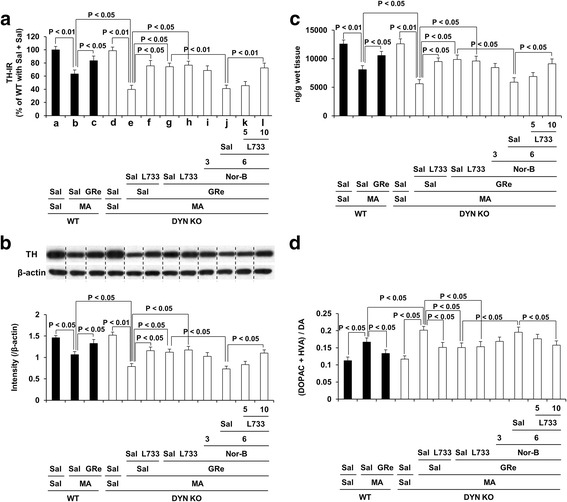
Role of κ-opioid and neurokinin 1 receptors in GRe-mediated dopaminergic neuroprotection. Effects of neurokinin 1 receptor antagonist L-733,060 on κ-opioid receptor antagonist Nor-B-mediated pharmacological activity in response to effects of GRe against changes in tyrosine hydroxylase-immunoreactivity (TH-IR; **a**), TH expression (**b**), dopamine level (**c**), and dopamine turnover rate (**d**) induced by MA in the striatum of DYN KO mice. Sal = saline, MA = methamphetamine 35 mg/kg, i.p., GRe = ginsenoside Re 20 mg/kg, i.p., Nor-B = Nor-binaltorphimine 3 or 6 mg/kg, i.p., L733 = L-733,060 5 or 10 mg/kg, i.p., WT = wild type, DYN KO = prodynorphin knockout. Each value is the mean ± SEM of six animals (one-way ANOVA followed by Fisher’s LSD pairwise comparisons)

MA-induced decrease (*p* < 0.01) in TH-immunoreactivity (TH-IR) of WT mice. This decrease was more pronounced (*p* < 0.05) in prodynorphin KO mice than in WT mice (Fig. 9a and Additional file 1: Figure S8). This decrease in prodynorphin KO mice was significantly protected by treatment with GRe (*p* < 0.05) or L-733,060 (*p* < 0.05). L-733,060 did not exhibit any additive effects against neuroprotective effects of GRe. Nor-B (6 mg/kg, i.p.) significantly counteracted (*p* < 0.01) the protective effects of GRe. Counteraction by Nor-B was significantly inhibited (*p* < 0.01) by L-733,060 (10 mg/kg, i.p.). Result of TH-IR paralleled that of TH expression and dopamine level, respectively (Fig. 9b, c). Consistently, increase (*p* < 0.05) in dopamine turnover rate was more pronounced in prodynorphin KO than in WT mice. Although GRe or L-733,060 attenuated this increase (*p* < 0.05), L-733,060 did not show any additional effects against dopaminergic neuroprotective activity of GRe. Nor-B treatment significantly counteracted (*p* < 0.05) neuroprotective effects of GRe. L-733,060 treatment inhibited (*p* < 0.05) against the counteraction induced by Nor-B (Fig. 9d).

The effects of Nor-B on the protective activity of GRe against dopaminergic impairments induced by MA in WT were shown in Additional file 1: Figure S9.

## Discussion

**Fig. 10:**
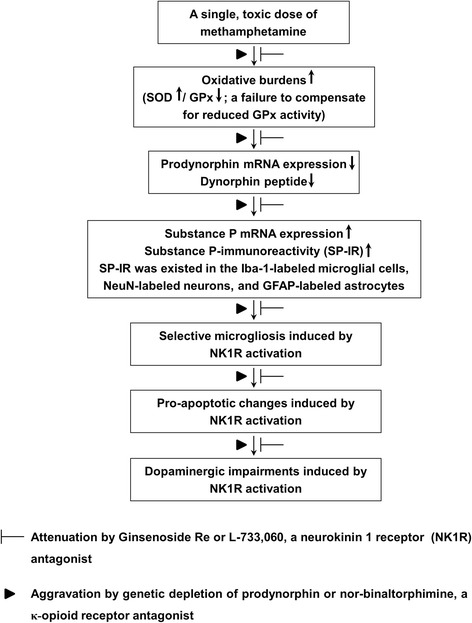
A schematic depiction ginsenoside Re (GRe)-mediated dopaminergic neuroprotective effects against MA insult. Treatment with a toxic dose of MA resulted in initial oxidative burdens, a perturbed redox status (a failure to compensate for reduced GPx) followed by the decreases in dynorphin level and prodynorphin mRNA expression. This decrease led to imbalance between dynorphin and substance P and to enhance substance P mRNA and substance P-immunoreactivity (SP-IR). At that time, SP-IR was co-localized in the Iba-1-immunoreactive microglia, NeuN-immunoreactive neurons, and GFAP-immunoreactive astrocytes. More importantly, GRe did not affect MA-induced astrocytic expression (i.e., GFAP expression), but GRe significantly attenuated against MA-induced microgliosis (i.e., Iba-1-immunoreactive-immunoreactivity and Iba-1 expression). Neurokinin 1 receptor activation, genetic depletion of dynorphin, or κ-opioid receptor antagonism (i.e., nor-binaltorphimine) potentiated this microgliosis, as well as pro-apoptotic changes. This morbid phenomenon contributed to dopaminergic impairment. Therefore, we propose that GRe attenuates MA-induced neurotoxicity via upregulation of prodynorphin-mediated κ-opioid receptor and downregulation of substance P-mediated NK1 receptor

Accumulating evidence has suggested that the positive effects of GRe may be due to its antioxidant and anti-inflammatory activities [30, 58]. Similarly, we observed that endogenous dynorphin plays a critical role in protecting dopaminergic neurons through its anti-inflammatory effects [3]. Here, we found that GRe modulated positive interaction between dynorphin and substance P to suppress oxidative stress and pro-inflammatory responses induced by MA. In line with previous reports [6, 7], we observed that NK1 receptor antagonism attenuated against MA-induced dopaminergic toxicity. However, NK1 receptor antagonist L-733,060 did not show any additional positive effect against neuroprotective activity by GRe, indicating that NK1 receptor is also one of the molecular pharmacological targets for GRe-mediated neuroprotective activity. Our result may be in line with a previous finding [23] that interaction between dynorphin and substance P is critical for modulating dopaminergic neurotoxicity. GRe treatment significantly attenuated MA-induced oxidative burdens, microglial activation, pro-apoptotic changes, and dopaminergic impairments via positive modulation between dynorphin-mediated κ-opioid receptor and substance P-mediated NK1 receptor (Fig. 10).

In this study, we selected a neurotoxic model induced by MA (35 mg/kg, i.p.), because this acute toxic model efficiently induces neuronal pro-apoptosis in the mouse striatum [53, 59]. Our recent reports indicated that a single, high dose of MA selectively induces proapoptosis in the striatum of mice [55, 60]. This range of MA dose also induces the loss of striatal dopamine terminal markers, such as dopamine transporter, tyrosine hydroxylase, and tissue dopamine content [61–63].

Dynorphin is the major posttranslational product of the prodynorphin gene and the presumed endogenous ligand for the κ-opioid receptor [34]. El Daly et al. [64] found that the systemic administration of a selective κ-opioid receptor agonist U69593 attenuated reduction in presynaptic dopamine neuronal function in response to repeated methamphetamine administration. Consistently, our finding indicated that κ-opioid receptor plays a protective role in dopaminergic neurodegeneration induced by a toxic dose of MA. κ-opioid receptor activation attenuates the initiation and long-term expression of sensitization to the locomotor-activating effects of cocaine [65–68]. Earlier studies have shown that κ-opioid receptor agonists modulate dopaminergic neurotransmission in the SN, neostriatum, and the mesolimbic system [69–72]. Administration of dynorphin (1–13), the postulated endogenous ligand for the κ-opioid receptor, also attenuated the development of D-amphetamine-induced behavioral sensitization [73]. Therefore, it is possible that κ-opioid receptor agonist might be important for positive dopaminergic modulation.

In this study, treatment with MA resulted in significant and constant increases in SOD activity in the dorsal striatum (> ventral midbrain > NAc) of mice, but did not involve a concomitant increase in GPx activity. Increased SOD activity can lead to an accumulation of H_2_O_2_, which in the absence of simultaneous increases in the activity of GPx, could increase Fenton reactions leading to the stimulation of ROS and lipid peroxidation/protein oxidation that result in irreversible cellular damage [74, 75]. In contrast, increased catalase activity in MA-treated wild-type mice could be an adaptive response to higher levels of H_2_O_2_ generated by inhibition of GPx activity, the brain has low-level catalase activity and only moderate amounts of SOD and GPx [75, 76]. Our observation of increased ROS, and lipid peroxidation/protein oxidation products implies that GPx activity, rather than increased SOD, modulates these endpoints. Furthermore, significant elevation of SOD activity in the early stages of MA insult in mice could be a response to the enhanced superoxide generated during MA-induced neurotoxicity [43, 77]. In other words, increased activity of SOD as well as decreased GPx activity in prodynorphin KO mice may be responsible for accumulating H_2_O_2_ levels, which could activate oxidative burdens. Therefore, the protective effect against MA-induced dopaminergic deficits in the presence of GRe might reflect an upregulation of dynorphin/ κ-opioid receptor as well as down-regulation of substance P/NK1 receptor.

GRe exerts an antioxidant potential, mitochondrial restoration, and anti-apoptosis via protein kinase C δ (PKCδ) inhibition in SH-SY5Y human neuroblastoma cells [58]. Shin et al. [30] also demonstrated that GRe rescued MA-induced oxidative damage, mitochondrial dysfunction, microglial activation, and dopaminergic degeneration by inhibiting PKCδ gene in mice. Previous studies have reported that substance P facilitated the phosphorylation of PKCδ in rat parotid acinar cells [78]. Moreover, it has been reported that PKCδ plays an important role in substance P-induced pro-inflammatory signaling in human colonocytes [79]. Substance P-induced PKCδ activation and its downstream signaling pathway are dependent on NK1 receptor [80]. Wang et al. [15] also identified PKCδ as a downstream signal that bridges substance P-mediated NK1 receptor activation and NOX2 activation in microglia. Therefore, it is plausible that GRe modulates molecular interaction between substance P and PKCδ against MA insult.

Substance P is considered the prototype of the tachykinin family and is encoded by the pre-protachykinin-A gene. Substance P is the natural ligand that displays the highest affinity for the NK1 receptor [6]. Earlier studies have found that multiple administrations of MA elevated the levels of pre-protachykinin-A mRNA and substance P within striatonigral neurons, suggesting that exposure to MA augments tachykinin neurotransmission in the striatonigral pathway [81–83]. High levels of substance P are present in the SN, where it binds to NK1 receptors expressed on dopaminergic neurons [5]. Consistently, we observed that treatment with MA resulted in a significant increase in substance P-immunoreactivity. Previous research indicated that substance P binding at NK1 receptors expressed on microglia and astrocytes may directly result in the activation of these glial cells in the central nervous system [84, 85]**.**

In this study, we observed that substance P localized with neurons, astrocytes, or microglia after MA treatment. In addition, treatment with MA resulted in increases in GFAP and Iba-1 expression. The increase in Iba-1 expression was attenuated by GRe or NK1 receptor antagonist L-733,060, which was consequently counteracted by κ-opioid receptor antagonist Nor-B. However, L-733,060, but not GRe, affected GFAP expression induced by MA. This finding supported previous study [15], suggesting that signaling events mediated by GRe, dynorphin, or substance P are specific to microglial cells.

Earlier study indicated that substance P and dynorphin co-exist extensively in specific populations of striatal projection neurons, indicating a correlation between two peptides [86]. Endogenous substance P potentiates immunological activation of microglia induced by lipopolysaccharide (LPS) or MPTP in the SN, suggesting the critical role of substance P as an inflammatory mediator in dopaminergic neurodegeneration [13, 14]. Block et al. [23] demonstrated a tightly regulated mechanism governing microglia-derived oxidative stress, in which the neuropeptide balance of dynorphin and substance P is critical to dopamine neuron survival. Substance P (10^− 13^–10^− 14^ M) is selectively toxic to dopaminergic neurons in the presence of microglia, which was significantly protected by dynorphin. In line with previous research [23], here, we proposed that the interaction between dynorphin and substance P may be essential for modulating dopaminergic toxicity induced by a single, high dose of MA (35 mg/kg, i.p.), indicating critical roles of κ-opioid receptor and NK1 receptor in modulating microglial activation.

Dopaminergic neurons in the SN are more susceptible to oxidative and inflammatory insults than neurons in other regions [87–90]. This phenomenon may be related to a higher density of microglia in SN than in other brain regions [91]. Similarly, a strong substance P-like immunoreactivity [92–94] has been shown in SN, and substance P is considered a likely neurotransmitter [95] which excites neurons in SN [96]. Wang et al. [15] proposed that substance P released from the nerve terminal of striatonigral projections may contribute to the higher microglial densities in the SN through the induction of proliferation and/or through chemotaxic recruitment of microglia. This is because the disproportionately higher density of microglia and potential substance P itself in the SN may contribute to the increased susceptibility of nigral dopaminergic neurons to regional neuroinflammation [91, 97].

It has been well-recognized that macrophages/microglia play different roles in tissue repair or damage in response to CNS injury. Microglia in the brain have classically activated M1 phenotype or alternatively activated M2 phenotype, depending on the inflammatory conditions of the local microenvironment. MA treatment enhanced the mRNA expression of M1 markers (CD16, CD32, and CD86), while those of M2 markers (arginase 1 and CD206) were decreased [3]. Endogenous dynorphin depletion accelerated microglial differentiation to the M1 phenotype after MA, suggesting that dynorphin may serve to dampen this neuroinflammatory process [3]. Importantly, treatment with GRe resulted in a significant attenuation against MA-induced increase in mRNA expression of M1 markers, indicating that GRe possesses an anti-inflammatory effect [30].

Microgliosis has been traditionally considered to play a passive role in the removal of dead or damaged neurons and debris by phagocytosis [3]. However, it is clear that microglial cells are reactivated during microgliosis, and further exacerbate neurodegeneration under severe inflammatory conditions [3]. It could be speculated that the balance between dynorphin and substance P appears to be critical for microglial activation and long-term survival of nigrostriatal dopaminergic neurons [23, 98]. Parallel with inducing microgliosis, MA can cause neuronal proapoptosis, in addition to terminal degeneration, by increasing caspase activity [55, 60, 99], current study].

Our earlier in vitro studies [98, 100] did not support our current in vivo study. Kong et al. [100] demonstrated that ultra-low concentrations (10^− 12^–10^− 16^ M) of dynorphin A-(1–8) significantly inhibited the LPS-induced production of NO or TNF-α in mixed glia cultures. The inhibitory effects of dynorphin A-(1–8) were not blocked by κ-opioid receptor antagonist Nor-B. Similarly, LPS-induced neurotoxicity in rat midbrain neuron-glia cultures was significantly reduced by treatment with ultra-low concentrations (10^− 13^–10^− 15^ M) of dynorphin A (1–17), but not by U50488, a synthetic κ-opioid receptor agonist [98], suggesting that dynorphin-mediated protective activity in vitro is not dependent on κ-opioid receptor. This disparity between our in vivo finding and previous in vitro reports remains to be fully elucidated.

It is well known that NAc is more resistant to MA toxicity [101], but the mechanism underlying the regional heterogeneity of MA has not yet been fully elucidated. The dopamine transporter (DAT) is believed to be an essential component of pathway leading to MA neurotoxicity in the striatum [102]. Supporting this notion, the basal level of DAT binding sites is higher in striatum than in NAc [6]. In addition, it was reported that a toxic dose of MA caused the delayed increase of glutamate release only in the striatum (caudate putamen), but not in the NAc [103]. A single injection of amphetamine or MA increased mRNA levels of preprotachykinin-A, along with preproenkephalin and preprodynorphin (encoding the neuropeptides substance P, enkephalines, and dynorphin, respectively) in the neostriatum with far greater elevation in caudate putamen than in NAc [104, 105], suggesting the differential actions of peptide systems to MA in brain regions. Observed regional differences between the caudate putamen and the NAc may reflect the existence of different mechanisms of MA-induced toxicity operating in these brain regions [6]. In addition, if nigrostriatal degeneration had occurred, MA treatment might activate microglial activation in the SN. However, MA-induced microglial activation in SN was much less than in striatum (Additional file 1: Figure S5), suggesting that MA-induced striatal burdens did not progress to ventral midbrain. It was demonstrated that the extent of toxicity within the ventral midbrain was greater in the SN pars compacta then in the reticulate and ventral tegmental area [106]. Rather, it is likely that the preservation of cellular integrity of ventral midbrain is a critical factor that allows for the recovery of the dopaminergic system [107]. Consistently, whole ventral midbrain is also resistant to MA toxicity in our experimental condition (please refer to Table 1, Fig. 8, and Additional file 1: Figure S1).

GRe is a main ginsenoside and an important ingredient in ginseng leaf, berry, and root [24, 35, 36, 108]. Ginseng root has been mainly used as herbal medicine, whereas other parts have been considered by-products or minor products. Earlier reports indicated that GRe is more abundant in the leaf, berry, and flower bud than in the root [35, 36, 109], reflecting possible pharmaco-economical advantages of GRe in terms of developing natural/drug resources.

## Conclusions

Our findings suggest that GRe exerts antioxidant, anti-inflammatory, and anti-apoptotic potentials against MA-induced dopaminergic neurotoxicity, and that interactive modulation between dynorphin and substance P is essential for GRe-mediated dopaminergic neuroprotection. The promising efficacy of GRe in response to MA insult may be helpful for developing therapeutic interventions for disorders of dopaminergic degeneration, although additional evidence should be obtained.

## Additional files

Additional file 1: Figure S1. MA-induced changes in dynorphin level in nucleus accimbens (NAc) and ventral midbrain in mice. **Figure S2.** MA-induced changes in substance P immunodistribution in the striatum and substantia nigra. **Figure S3.** Role of κ-opioid receptor in GRe-mediated modulation in substance P mRNA expression. **Figure S4.** Role of κ-opioid receptor in GRe-mediated antioxidant potentials. **Figure S5.** MA-induced changes in Iba-1 immunoreactive microglial cells in the striatum and substantia nigra. **Figure S6.** Role of κ-opioid receptor in GRe-mediated anti-microglial potentials. **Figure S7.** Role of κ-opioid receptor in GRe-mediated anti-apoptotic potentials. **Figure S8.** Role of κ-opioid and neurokinin 1 receptors in GRe-mediated attenuation on the loss of TH-immunoreactivity (TH-IR). **Figure S9.** Role of κ-opioid receptor in GRe-mediated dopaminergic neuroprotection. (DOCX 18 kb)

## Abbreviations

ANOVA: Analysis of variance
BSA: Bovine serum albumin
CNS: Central nervous system
DA: Dopamine
DAergic: Dopaminergic
DCF: Dichlorofluorescin
DCFH-DA: 2′, 7′-dichlorofluorescin diacetate
DNPH: 2, 4-dinitrophenylhydrazine
DYN KO: Prodynorphin knockout mice
EDTA: Ethylenediaminetetraacetic acid
GFAP: Glial fibrillary acidic protein
GRe: Ginsenoside Re
HNE: 4-hydroxynonenal
Iba-1: Ionized calcium binding adaptor molecule 1
LPS: Lipopolysaccharides
MA: Methamphetamine
MPTP: 1-methyl-4-phenyl-1, 2, 3, 6-tetrahydropyridine
NK1 receptor: Neurokinin 1 receptor
Nor-B: Nor-binaltorphimine
PBS: Phosphate-buffered saline
PD: Parkinson’s disease
PKC: Protein kinase C
ROS: Reactive oxygen species
RT-PCR: Reverse transcription and polymerase chain reaction
SN: Substantia nigra
TH: Tyrosine hydroxylase
TH-IR: TH-immunoreactivity
WT: Wild type
