# Supplementary material for: Ginsenoside Re protects methamphetamine-induced dopaminergic neurotoxicity in mice via upregulation of dynorphin-mediated κ-opioid receptor and downregulation of substance P-mediated neurokinin 1 receptor
Source: J Neuroinflammation. 2018 Feb 21;15:52. doi: 10.1186/s12974-018-1087-7 (PMC5822489; doi:10.1186/s12974-018-1087-7)
Supplement: Supplementary file 1 — MA-induced changes in dynorphin level in nucleus accimbens (NAc) and ventral midbrain in mice. Figure S2. MA-induced changes in substance P immunodistribution in the striatum and substantia nigra. Figure S3. Role of κ-opioid receptor in GRe-mediated modulation in substance P mRNA expression. Figure S4. Role of κ-opioid receptor in GRe-mediated antioxidant potentials. Figure S5. MA-induced changes in Iba-1 immunoreactive microglial cells in the striatum and substantia nigra. Figure S6. Role of κ-opioid receptor in GRe-mediated anti-microglial potentials. Figure S7. Role of κ-opioid receptor in GRe-mediated anti-apoptotic potentials. Figure S8. Role of κ-opioid and neurokinin 1 receptors in GRe-mediated attenuation on the loss of TH-immunoreactivity (TH-IR). Figure S9. Role of κ-opioid receptor in GRe-mediated dopaminergic neuroprotection. (DOCX 18 kb) [file 12974_2018_1087_MOESM1_ESM.docx]

**Supplemental Information**

**Supplemental Results**

**MA-induced changes in dynorphin level the nucleus accumbens (NAc) and ventral midbrain of wild type (WT) mice.**

As shown in Supplementary Fig. S1, MA treatment significantly decreased dynorphin level in the NAc and ventral midbrain 6 h (NAc, p < 0.05 vs. saline; Ventral midbrain, p < 0.01 vs. saline), 12 h (NAc, p < 0.05 vs. saline; Ventral midbrain, p < 0.01 vs. saline), and 1 d (NAc, p < 0.05 vs. saline; Ventral midbrain, p < 0.01 vs. saline) post-MA in WT mice.

**Substance P-immunoreactivity (SP-IR) in the striatum and substantia nigra (SN) after MA administration**

As shown in Supplemental Fig. S2A-C, MA (35 mg/kg, i.p.) did not significantly affect SP-IR in the striatum of WT mice. However, treatment with MA (35 mg/kg, i.p.) resulted in a significant increase (p < 0.01) in SP-IR in the SN.

**Effects of κ-opioid receptor antagonist, nor-binaltorphimine (Nor-B) on the pharmacological activity of ginsenoside Re (GRe) against MA-induced increase in substance P mRNA expression in the substantia nigra of WT mice**

As presented in Supplemental Fig. S3, GRe significantly attenuated (p < 0.05) increase in substance P mRNA expression induced by MA, which was consequently counteracted (p < 0.05) by Nor-B (6 mg/kg, i.p.). In addition, Nor-B itself potentiated (p < 0.05) MA-induced increase in substance P mRNA level.

**Effects of κ-opioid receptor antagonist, nor-binaltorphimine (Nor-B) on the antioxidant effect of ginsenoside Re (GRe) against MA insult in the striatum of WT mice**

As presented in Supplemental Fig. S4A-C, Nor-B counteracted (ROS, HNE, or protein carbonyl level: p < 0.05, respectively) antioxidant effects of GRe in a dose-related manner. Nor-B facilitated oxidative damage (ROS, HNE, or protein carbonyl level: p < 0.05, respectively) induced by MA.

**Iba-1-IR in the striatum and substantia nigra (SN) after MA administration**

As shown in Supplemental Fig. S5A-C, MA-induced increase in Iba-1-IR in the striatum (p < 0.01)(A, C) and SN (p < 0.05)(B, C) of WT mice. This increase is more pronounced (p < 0.05) in the striatum than in the SN (C).

**Effects of κ-opioid receptor antagonist, nor-binaltorphimine (Nor-B) on the pharmacological activity of ginsenoside Re (GRe) against MA-induced microgliosis in the striatum of WT mice**

As presented in Supplemental Fig. S6A and B, GRe significantly attenuated MA-induced increases in Iba-1-IR (p < 0.05) and Iba-1 expression (p < 0.05). Nor-B (6 mg/kg, i.p.) facilitated increases in Iba-1-IR (p < 0.05) and Iba-1 expression (p < 0.05) induced by MA. Nor-B also counteracted (p < 0.01) GRe-mediated attenuation against microglial activation induced by MA.

**Effects of κ-opioid receptor antagonist, nor-binaltorphimine (Nor-B) on the pharmacological activity of ginsenoside Re (GRe) against MA-induced pro-apoptotic changes in the striatum of WT mice**

As presented in Supplemental Fig. S7A-C, MA-induced increases in Bax (p < 0.05) and cleaved caspase-3 (p < 0.05) expression was significantly attenuated (p < 0.05) by GRe treatment. Consistently, MA significantly decreased (p < 0.05) Bcl-2 expression. This decrease was significantly attenuated (p < 0.05) by GRe. Nor-B facilitated the increases in Bax (p < 0.05) and cleaved caspase-3 (p < 0.05) expression and decrease in Bcl-2 expression (p < 0.05). Moreover, the attenuation by GRe was significantly counteracted (p < 0.05) by Nor-B.

**Effects of neurokinin 1 receptor antagonist L-733,060 on κ-opioid receptor antagonist Nor-B-mediated pharmacological activity in response to effects of GRe against the decrease in tyrosine hydroxylase-immunoreactivity (TH-IR) induced by MA in the striatum of prodynorphin KO mice.**

As shown in Supplementary Fig. S8, the representative photomicrographs support the results of Fig. 9A.

**Effects of κ-opioid receptor antagonist, nor-binaltorphimine (Nor-B) on the pharmacological activity of ginsenoside Re (GRe) against MA-induced dopaminergic impairments in the striatum WT mice**

As shown in Supplemental Fig. S9-D, MA-induced decrease (p < 0.05) in TH-IR, which was significantly attenuated (p < 0.05) by GRe. Nor-B (6 mg/kg, i.p.) significantly facilitated (p < 0.05) MA-induced decrease in TH-IR. Result of TH-IR paralleled that of TH expression and dopamine level, respectively. Consistently, GRe significantly attenuated (p < 0.05) against MA-induced increase in dopamine turnover rate. This increase was accelerated (p < 0.05) by Nor-B. Nor-B counteracted GRe-mediated dopaminergic neuroprotective effects against MA-induced decreases in TH-IR (p < 0.05), TH expression (p < 0.05), and dopamine level (p < 0.05), and increase in dopamine turnover rate (p < 0.05).

**Supplemental Figure Legends**

**Figure S1. MA-induced changes in dynorphin level in nucleus accimbens (NAc) and ventral midbrain in mice.** Sal = saline. MA = methamphetamine 35 mg/kg, i.p. Each value is the mean ± SEM of six animals (one-way ANOVA followed by Fisher’s LSD pairwise comparisons).

**Figure S2. MA-induced changes in substance P immunodistribution in the striatum and substantia nigra.** Effect of a single, high dose of MA on substance P-immunoreactivity (SP-IR) in the striatum (A, C) and substantia nigra (B, C) of mice. Sal = saline. MA = methamphetamine 35 mg/kg, i.p. Each value is the mean ± SEM of six animals (one-way ANOVA). Scale bar = 200 µm.

**Figure S3.** **Role of κ-opioid receptor in GRe-mediated modulation in substance P mRNA expression.** Effects of κ-opioid receptor antagonist Nor-B on GRe-mediated activity against MA-induced increase in substance P mRNA expression in the substantia nigra of WT mice. Sal = saline. MA = methamphetamine 35 mg/kg, i.p. GRe = ginsenoside Re 20 mg/kg, i.p. Nor-B = Nor-binaltorphimine 3 or 6 mg/kg, i.p. Each value is the mean ± SEM of six animals (one-way ANOVA followed by Fisher’s LSD pairwise comparisons).

**Figure S4. Role of κ-opioid receptor in GRe-mediated antioxidant potentials.** Effects of κ-opioid receptor antagonist Nor-B on antioxidant effect of GRe against ROS formation (A), HNE (B), and protein carbonyl (C) levels induced by MA in the striatum of WT mice. Sal = saline. MA = methamphetamine 35 mg/kg, i.p. GRe = ginsenoside Re 20 mg/kg, i.p. Nor-B = Nor-binaltorphimine 3 or 6 mg/kg, i.p. Each value is the mean ± SEM of six animals (one-way ANOVA followed by Fisher’s LSD pairwise comparisons).

**Figure S5. MA-induced changes in Iba-1 immunoreactive microglial cells in the striatum and substantia nigra.** Striatal Iba-1-immunoreactivity (Iba-1-IR) (A, C) is significantly higher than nigral Iba-1-IR (B, C) after MA (35 mg/kg, i.p.) administration. Sal = saline. MA = methamphetamine 35 mg/kg, i.p. Each value is the mean ± SEM of six animals (two-way ANOVA followed by Fisher’s LSD pairwise comparisons). Scale bar = 200 µm.

**Figure S6.** **Role of κ-opioid receptor in GRe-mediated anti-microglial potentials.** Effects of κ-opioid receptor antagonist Nor-B on GRe-mediated attenuation against MA-induced increases in Iba-1-IR (A) and Iba-1 (B) expression (B) in the striatum of WT mice. Sal = saline. MA = methamphetamine 35 mg/kg, i.p. GRe = ginsenoside Re 20 mg/kg, i.p. Nor-B = Nor-binaltorphimine 3 or 6 mg/kg, i.p. Each value is the mean ± SEM of six animals (one-way ANOVA followed by Fisher’s LSD pairwise comparisons). Scale bar = 100 µm.

**Figure S7.** **Role of κ-opioid receptor in GRe-mediated anti-apoptotic potentials.** Effects of κ-opioid receptor antagonist Nor-B on GRe-mediated activity against MA-induced changes in Bax (A), cleaved caspase-3 (B), and Bcl-2 (C) expression in the striatum of WT mice. Sal = saline. MA = methamphetamine 35 mg/kg, i.p. GRe = ginsenoside Re 20 mg/kg, i.p. Nor-B = Nor-binaltorphimine 3 or 6 mg/kg, i.p. Each value is the mean ± SEM of six animals (one-way ANOVA followed by Fisher’s LSD pairwise comparisons).

**Figure S8. Role of κ-opioid and neurokinin 1 receptors in GRe-mediated attenuation on the loss of TH-immunoreactivity (TH-IR).** Effects of neurokinin 1 receptor antagonist L-733,060 on κ-opioid receptor antagonist Nor-B-mediated pharmacological activity in response to effects of GRe against reduction in TH-IR induced by MA in the striatum of DYN KO mice. Sal = saline, MA = methamphetamine 35 mg/kg, i.p., GRe = ginsenoside Re 20 mg/kg, i.p., Nor-B (3) or Nor-B (6) = Nor-binaltorphimine 3 or 6 mg/kg, i.p., L733 (5) or L733 (10) = L-733,060 5 or 10 mg/kg, i.p., WT = wild-type, DYN KO = prodynorphin knockout. Each value is the mean ± SEM of six animals (one-way ANOVA followed by Fisher’s LSD pairwise comparisons). Scale bar = 1 mm.

**Figure S9.** **Role of κ-opioid receptor in GRe-mediated dopaminergic neuroprotection.** Effects of κ-opioid receptor antagonist Nor-B on GRe-mediated activity against MA-induced changes in tyrosine hydroxylase-immunoreactivity (TH-IR; A), TH expression (B), dopamine level (C), and dopamine turnover rate (D) in the striatum of WT mice. Sal = saline. MA = methamphetamine 35 mg/kg, i.p. GRe = ginsenoside Re 20 mg/kg, i.p. Nor-B = Nor-binaltorphimine 3 or 6 mg/kg, i.p. Each value is the mean ± SEM of six animals (one-way ANOVA followed by Fisher’s LSD pairwise comparisons). Scale bar = 1 mm
